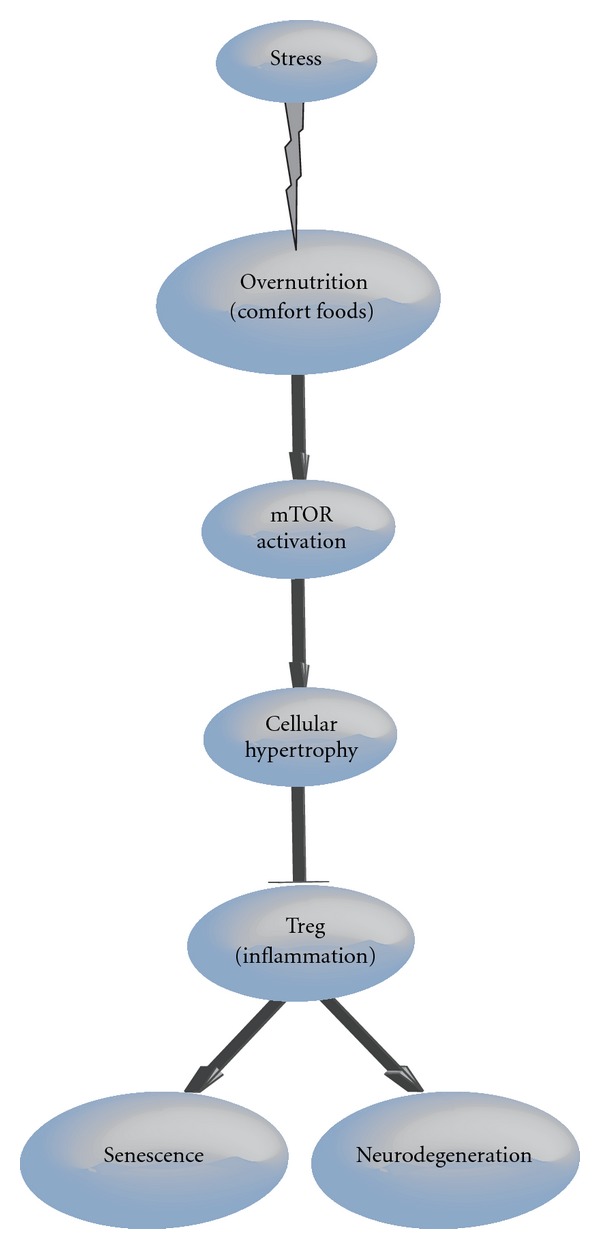# Alzheimer's Disease Promotion by Obesity: Induced Mechanisms—Molecular Links and Perspectives

**DOI:** 10.1155/2012/986823

**Published:** 2012-06-04

**Authors:** Rita Businaro, Flora Ippoliti, Serafino Ricci, Nicoletta Canitano, Andrea Fuso

**Affiliations:** ^1^Medico-Surgical Sciences and Biotechnologies, Sapienza University of Rome, Corso della Repubblica 79, 04100 Latina, Italy; ^2^Department of Experimental Medicine, Sapienza University of Rome, Viale Regina Elena 324, 00161 Rome, Italy; ^3^Department of Anatomy, Histology, Forensic Medicine and Orthopedics, Sapienza University of Rome, Viale Regina Elena 336, 00161 Rome, Italy; ^4^Department of Surgery “P. Valdoni,” Sapienza University of Rome, Via A. scarpa 14, 00161 Rome, Italy

## Abstract

The incidence of AD is increasing in parallel with the increase in life expectancy. At the same time the prevalence of metabolic syndrome and obesity is reaching epidemic proportions in western populations. Stress is one of the major inducers of visceral fat and obesity development, underlying accelerated aging processes. Adipose tissue is at present considered as an active endocrine organ, producing important mediators involved in metabolism regulation as well as in inflammatory mechanisms. Insulin and leptin resistance has been related to the dysregulation of energy balance and to the induction of a chronic inflammatory status which have been recognized as important cofactors in cognitive impairment and AD initiation and progression. The aim of this paper is to disclose the correlation between the onset and progression of AD and the stress-induced changes in lifestyle, leading to overnutrition and reduced physical activity, ending with metabolic syndrome and obesity. The involved molecular mechanisms will be briefly discussed, and advisable guide lines for the prevention of AD through lifestyle modifications will be proposed.

## 1. Alzheimer's Disease (AD)

Alzheimer's disease (AD) is the most common cause of dementia, accounting for 60–80% of cases, although there is growing awareness that AD is often confused with other causes of dementia. According to estimates by 2006, approximately 33.9 million people worldwide have AD [1], and Alzheimer's Association estimates 5 to 3 million people in the US have the disease [2]. It is foreseen that the prevalence will nearly triple [1] or increase from three to four times, according to other studies, in the next 40 years due to demographic changes and a longer life expectancy [3]. Among the sixty year olds, those who show a higher prevalence are North Americans (6.4%) and Western Europeans (5.4%). For the rest there is 4.9% in Latin America. It is to outline that incidence is likely to increase in proportion to the aging population, which by 2030 would increase by 250% in industrialized countries. Previous data show that rates of dementia increase exponentially with age [4]. The incidence of dementia doubles every 5 years, from 0.66/100 persons aged 70 to 74 years to 11.30/100 persons for those aged 90 or more.

A bulk of studies has provided evidence to support the role of obesity as a risk factor for AD development and the possible role of psychosocial factors (e.g., professional achievements, stimulant mental activities, social engagement, and physical activity) as protective factors.

### 1.1. Stress in Modern Societies That Influences AD

“Lifestyle has dramatically changed in modern societies and social psychological stress is ubiquitous and universally pervasive. In modern life, statistics show powerful effects of early-life stress, concurrent chronic stress, and socioeconomic status with sociopolitical system have a potent effect on the burden of chronic disease” [5].

 Increasing evidence has been accumulating about the role of stress as an important challenge to the onset and progression of AD [6]. The heterogeneous nature of AD is only partly explained by the brain's propensity to accumulate aberrantly processed, misfolded, and aggregated oligomeric structural proteins, including amyloid-*β* peptides and hyperphosphorylated tau.

## 2. Globesity

Obesity and the metabolic syndrome are challenging public health issues since their prevalence in Western populations has reached epidemic proportions. In 1997 the World Health Organization (WHO) stated that “…obesity should now be regarded as one of the greatest neglected public health problems of our time….” During the last four decades the world has experienced an epidemic of overweight individuals and the WHO has predicted a “globesity epidemic” with more than 1 billion adults being overweight and at least 300 million of these being clinically obese [7]. In the United States approximately 65% of adults are overweight or obese [8], and almost half of Italian men and about 1 of 3 Italian women are overweight or obese [9].

The excess of adiposity is an established risk factor for the development of cardiovascular diseases, type 2 diabetes, and hypertension, all characterized by resistance to insulin-mediated glucose disposal. Insulin resistance and the compensatory hyperinsulinemia associated with insulin resistance have been shown to be independent predictors of all three clinical syndrome [10]. Several studies have reported that obesity, generally defined as a body mass index BMI > 30, increases the risk of disease and all-cause mortality and reduces life expectancy [11]. Caucasian individuals, who reached a BMI > 40 between the ages of 20–29 years, could expect a reduction in remaining years of life expected by approximately 6 and 12 years, respectively [11, 12]. Obesity has not only been linked to reduced life expectancy but also to accelerated aging, as demonstrated by obese women having telomeres that were 240 bp shorter compared to lean women of similar age [13].

 Many factors influence the onset of obesity, including genetic, environmental, socioeconomical, behavioral, and/or psychological factors. The main cause that leads to the development of obesity is a positive energy balance, which consists in imbalance between energy intake and expenditure, lasting for several years. Such a balance is regulated by a complex network of signals that connect the endocrine system with the central nervous system [14]. Overnutrition, leading to obesity, impairs systemic metabolic homeostasis and is a metabolic stressor associated with intracellular organelles (e.g., the endoplasmic reticulum) stress. Starvation and malnutrition can impair immune function too [15].

Different kinds of stressors, including life stressful events, on the other hand, have been particularly linked to development of visceral obesity [16]. The hypothalamic-pituitary-adrenal axis and the central and peripheral components of the autonomic nervous system constitute the two main vital stress-system functions [5]. States of over- or under-nutrition may impair the crosstalk between metabolic and immune system, leading to the activation of the immune response and the development of a “low-grade systemic inflammation,” as confirmed by increasing circulating levels of proinflammatory cytokines, adipokines and other inflammatory markers detected in obese subjects. Activation of the immune response in obesity is mediated by specific signaling pathways, with Jun N-terminal kinase and IkappaB kinase beta/nuclear factor kappa-light-chain-enhancer of activated B cells being the most studied. It is known that the above events modify insulin signaling and result in the development of insulin resistance [16].

### 2.1. Visceral Fat (VF) and Subcutaneous Fat (SF)

Increased body mass induces the formation of fat deposits in the visceral and subcutaneous structures [12]. Fat tissue is at present considered as an active endocrine organ with a high metabolic activity. It produces several mediators that are important in metabolism (adipokines) and inflammation (cytokines). Many of these cytokines also referred to as “adipokines,” including leptin, TNF-*α*, IL-6, heparin-binding epidermal growth factor (HB-EGF), and vascular endothelial growth factor (VEGF) among others, may play an important role in many diseases by promoting angiogenesis, inflammation, cell proliferation and insulin resistance [12].

Activation of proinflammatory pathways and secretion of cytokines such as interleukin-6 (IL-6), plasminogen-activator inhibitor-1, and free fatty acids (FFA) have been suggested to produce insulin resistance [17, 18]. Fat accumulation in the abdominal area is considered one of the main risk factors for developing cardiovascular and metabolic diseases. This effect is probably depending on cytokines synthesized by visceral adipose tissue and released into the portal circulation, thereby reaching the liver, where they can trigger a series of inflammatory events, including greater FFA and glycerol release [19]. In particular the proinflammatory cytokine IL-6 regulates hepatic protein synthesis by evoking an acute phase response such as C-reactive protein (CRP) or serum amyloid-A. As reported by Libby and colleagues, the Quebec Heart Study has shown that obesity is associated with systemic inflammation, since there is a correlation between VF and the levels of CRP [20]. Studies in rodents and humans have revealed that body fat distribution, including VF, SF, and ectopic fat is critical for determining the risk posed by obesity [21].

VF secretes more cytokines than subcutaneous adipose tissue; in addition using either waist circumference and/or waist-to-hip ratio as a proxy of abdominal obesity, numerous studies have found that VF is a stronger risk factor for metabolic and cardiovascular diseases than body mass index (BMI) or other fat depots. Adipose tissue can produce several modulators of inflammation. This condition promotes the development of diabetes mellitus (DM) which is accompanied by an increased risk of both macrovascular and microvascular disease. The negative impact of obesity on cognitive function may be, at least in part, due to vascular defects, impaired insulin metabolism and signaling pathway or a defect in glucose transport mechanisms in brain. It is plausible to hypothesise that increased vascular dysfunction at the level of the brain may in turn affect memory function [22].

 In a study performed on male C57BL/6 mice fed a standard diet low in fat until the age of 6 weeks and then switched to a high fat diet for the following 15 or 21 weeks, it has been shown that obese mice exhibited a higher concentration of macrophages in visceral adipose tissue compared to lean animals [23].

VF is associated with a low-grade inflammation due to the increased secretion of numerous pro-inflammatory cytokines from adipocytes and their associated macrophages [12]. In this connection, our previous results showed that in obese children, the presence of a chronic low-grade inflammation corresponds to a shift to Th1-cytokine profile dominated by the production of IFN-gamma, accompanied by insulin resistance [24]. However, the molecular basis for the association between obesity and low-degree chronic inflammation is still unknown.

### 2.2. AD Can Be Considered a Metabolic Disease

Diabetes mellitus (DM) is a risk factor for nongenetic AD. Recent studies in humans indicate that insulin signaling is impaired in the AD brain [25]. Insulin is the hormone in charge of tissutal glucose uptake, it binds to specific receptors expressed on cell membranes and triggers the phosphorylation of cellular substrates. A switch from a tyrosine phosphorylation to a serine phosphorylation of the insulin receptor substrate (IRS) family of proteins impairs the metabolic activity of insulin leading to insulin resistance and type 2 diabetes. This alteration may be mediated by stress and inflammation, as shown by the effects of cytokines released by immune cells. Insulin receptors and insulin-sensitive glucose transporters have been detected at the level of the medial temporal region of the brain that supports memory formation, leading to hypothesize that insulin may be involved in maintaining normal cognitive function [22]. Moreover, AD is associated with cerebrovascular amyloid angiopathy in which an increased expression of RAGE (receptor for advanced glycation endproducts) was detected. Carbohydrate-derived advanced glycation endproducts (AGEs) have been implicated in the chronic complications of DM and have been reported to play an important role in the pathogenesis of AD [26]. They have been localized in AD brain and their presence was shown to be significantly increased in human post-mortem samples of AD with diabetes compared to AD without diabetes or nondemented controls [27]. RAGE has been found to be a specific cell surface receptor for amyloid *β*, thus potentially facilitating neuronal damage [28, 29].

A recent paper by de la Monte [30] discusses the direct relationship between impaired insulin/IGF signaling, increased amyloid-*β* precursor protein (A*β*PP) synthesis, and the increased accumulation of A*β* peptide in amyloid plaques, promoting neurodegeneration. The mechanism proposed by de la Monte is that brain insulin deficiency and resistance cause neuronal death due to trophic factor withdrawal, deficits in energy metabolism, and inhibition of insulin-responsive gene expression, including those required for acetylcholine homeostasis. It is known that insulin resistance increases with age and that normal blood glucose levels are maintained until the body is able to provide an adequate amount of insulin (hyperinsulinemia). As described above, peripheral insulin resistance is mediated by the inflammatory process that takes place within adipose tissue, enlarging abdominal fat in obese individuals. As a matter of fact, insulin resistance is a common finding in chronic inflammatory diseases and, in particular, it is believed that increased adipose-derived inflammatory cytokines induce a chronic inflammatory state that not only increases cardiovascular risk, but also antagonizes insulin signaling and mitochondrial function thereby impairing glucose homeostasis [31, 32].

### 2.3. Energy Metabolism Dysregulation

The decreased energy metabolism due to insulin resistance first and then to reduced glucose uptake impairs ATP production. Chronic inflammation, such as detected in obesity and stress conditions, implies a constant, although low-grade, activation of the immune system, as evidenced by the increased serum levels of proinflammatory cytokines in subjects suffering from chronic inflammatory diseases. In this connection, Straub et al. [33] reported that neuroendocrine pathways are involved in energy regulation: inflammation induces an increase in cortisol serum levels, by stimulating HPA axis and sympathetic nervous system (SNS), ending with sickness behavior, characterized by strong reduction of muscle, brain, and gut activity. Fat gain depends, inter alia, on a lack of physical activity which brings to muscle loss and increased leptin levels which, in turn, support muscle loss and fat gain, leading to cachectic obesity [33]. In this respect it is to remind that proinflammatory cytokines can disturb insulin receptor and IGF-1 receptor signaling [34] and that FFA induce insulin resistance [35].

**Figure 1:**
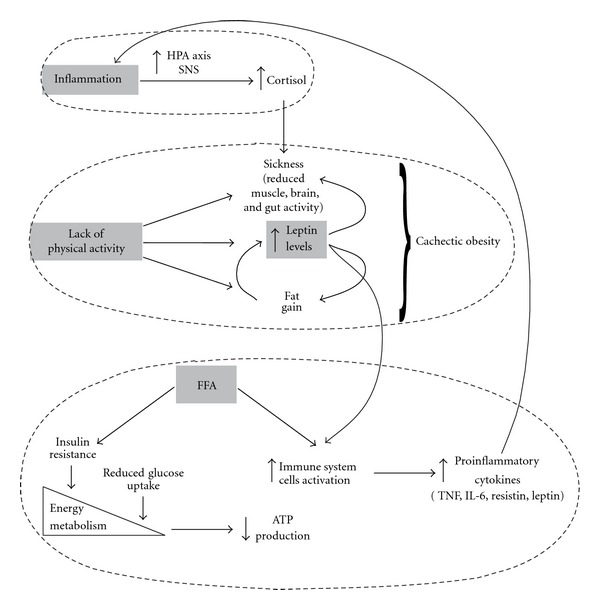
The complex and interconnected pathways linking stress, inflammation, obesity and energy metabolism.

As a result, insulin resistance produces a dysregulation of energy balance at the level of liver, adipose tissue, and muscle and, at the same time, favours the activated cells of the immune system since they do not become insulin resistant. Leptin, whose release is increased following the enlargement of fat, stimulates glucose uptake by immune cells and therefore their metabolic activity. In this way a vicious circle takes place, with a continuous release of proinflammatory adipokines such TNF, IL-6, resistin and leptin which contribute to maintain a chronic inflammatory state. Several inflammatory pathways have been shown to contribute to metabolic dysregulation at several levels, among them the modulation of insulin signalling is perhaps the most crucial, as it is a highly conserved and dominant metabolic pathway in nutrient and energy homeostasis. In addition to cytokines, many of the inflammatory signalling pathways that inhibit insulin receptor signalling are directly triggered by nutrients, such as circulating lipids. Other inflammatory pathways are induced by organelle stress owing to nutrient overload and processing defects and result in metabolic stress. These complexes connections are schematically depicted in Figure 1.

### 2.4. Obesity and Systemic Inflammation

Macrophage accumulation and the subsequent local inflammation are believed to result in numerous metabolic dysfunctions that accompany obesity, including systemic inflammation. Macrophages and adipocytes are closely related and share many functions: for example, they both secrete cytokines and can be activated by pathogen-associated components, such as lipopolysaccharide (LPS) [36]. Preadipocytes have been shown to transdifferentiate into macrophages, and transcriptional profiling has suggested that macrophages and pre-adipocytes are genetically related [37].

AD was recently added to the obesity-related diseases taking into account the release of inflammatory cytokines by activated macrophages in visceral adipose tissue. Several recent studies prospectively assessed the predictive value of elevated pro-inflammatory cytokines for the risk of developing AD in cognitively intact individuals or for aggravating AD symptoms in patients who were already diagnosed with the disease. Higher plasma levels of the inflammatory marker *α*1-antichymotrypsin and IL-6 [38], as well as higher spontaneous production of IL-1*β* or TNF-*α* by peripheral blood mononuclear cells [39], were found to be associated with increased future risk of AD in older individuals.

The Framingham Heart Study comprising male participants (age range 55–88 years) followed up over a period of 18 years revealed that obesity had an adverse effect on cognitive performance [40]. In agreement with this finding, Osher and Stern described that obesity may contribute to the reduction of cognitive skills observed in AD [41].

In the otherwise healthy older population, the combination of expansive waist circumference or BMI, with high systolic or diastolic blood pressure, was linked to a modest decrease in performance on tests of motor speed, manual dexterity, and executive function [42]. The association appeared to be so profound that the risk for AD increased by 36% for every BMI unit at the age of 70 years.

Crosstalk between lymphocytes and adipocytes can lead to immune regulation. Adipose tissue produces and releases a variety of proinflammatory and anti-inflammatory factors, including the adipokines leptin, adiponectin, resistin, and visfatin, as well as cytokines and chemokines, such as TNF-*α*, IL-6, monocyte chemoattractant protein 1 (MCP-1), and others. Proinflammatory molecules produced by adipose tissue have been implicated as active participants in the development of insulin resistance and increased risk of cardiovascular disease associated with obesity [43].

### 2.5. Cytokines, Adipose Tissue and AD

Immune system influences central nervous system through the release of cytokines targeting different brain districts. Cytokines mediate not only immune response but also neuron functions and survival [44]. They may originate from peripheral immune cells and reach the CNS by crossing the blood-brain barrier or may be directly produced within the CNS by neurons and glial cells [45]. Cytokines bind to specific cytokine receptors on neurons and glial cells thereby directly influencing brain function. Two main clusters of cytokines have been recognized, based on the specific T-helper cells producing them: type 1 helper cells, generally engaged in cellular immune response and type 2 helper cells involved in humoral immunity.

Cytokines are commonly classified in two categories: interleukin-1 (IL-1), tumor necrosis factor *α* (TNF-*α*), interferon*γ* (IFN*γ*), IL-12, IL-18 and granulocyte-macrophage colony stimulating factor (GM-CSF) are well characterized as pro-inflammatory cytokines whereas IL-4, IL-10, IL-13 and transforming growth factor-*β* (TGF-*β*) are recognized as anti-inflammatory cytokines.

### 2.6. Adipokines

The family of adipokines is continuously expanding and includes also INF*γ*, LIF (Leukemia Inhibiting Factor) and chemokines such as the MCP-1 and MIP-1 (Macrophagic Inflammatory Protein-1). TNF-*α* is secreted by the activated macrophages and by adipocytes and plays an important role in the defence of the host from infections and in the development of the Th-1 subpopulations; it is involved in the pathogenesis of autoimmune diseases such as the multiple sclerosis, rheumatoid arthritis, and I type diabetes. Patients affected by insulin resistance present increased TNF-*α* levels and mice with a TNF-*α* deficiency are protected by the insulin resistance-induced obesity.

Leptin is a typical adipokine produced in proportion to the amount of the body fat; indeed its levels are related to BMI. It has been shown that there is a direct link between leptin, leptin receptor, and activation of mTOR (mammalian target of rapamycin) [46]. Leptin and nutrients (i.e., amino acids and glucose) show pulsatile secretion in vivo and living cells continuously adjust their gene expression in response to the changing milieu that influences the energy status into the cells and modulates cell growth, proliferation, and differentiation. The maker of this mechanism is the mammalian target of rapamycin (mTOR), an evolutionarily conserved 289-kDa serine-threonine protein kinase that is inhibited by rapamycin [47]. Within this context, it has been hypothesized that leptin might act as an endogenous “sensing” factor that could act as a critical link among environment (availability of nutrients), metabolism, and immune responses [48]. Pro-inflammatory activity of leptin, that potentiates T helper 1 (Th1) immune responses, is due to decreased Treg cell proliferation [49, 50]. Matarese and colleagues showed that the leptin/mTOR signalling pathway influences Treg cell responsiveness according to the energy metabolism. High metabolic rate determines Treg cell hyporesponsiveness sustained by mTOR activation, whereas inhibition of mTOR with rapamycin enhances Treg cell proliferation and their anti-inflammatory activity [47]. Leptin-mTOR overexpression in freshly isolated Treg cells is responsible for their state of hyporesponsiveness. The hypothesis that a metabolic control of immune-mediated pathogenesis of obesity and obesity-related insulin resistance exist [51, 52], has recently reinforced the concept that metabolism and proliferation of lymphocytes can impact, at different levels, the control of inflammation, autoimmunity, and immune-mediated disorders [48, 53]. In all of these conditions, Treg cells have a high metabolic state, high ATP and mTOR activity and are unresponsive to regulatory action in immune/inflammatory response [54]. Blood leptin levels are directly correlated with adiposity [55, 56]. In the presence of excessive food intake, aggravated by psychological stress, the increase of leptin induces activation of mTOR that determines adverse effects on age-related diseases and inhibition of autophagy in the liver (lipophagy) which contributes to steatosis and lipid accumulation in VF [57]. In the hypothalamus, leptin inhibits food intake through mTOR activation, and mTOR inhibition with rapamycin prevents leptin-induced anorexia [58, 59].

In a condition of lack of food and consequent reduction of the body fat mass, low levels of leptin lead to a reduced metabolic waste to preserve the energy necessary to support the functions of vital organs such as heart, kidney, and brain; on the other hand, the finding of high levels of leptin in obese subjects has been interpreted as the result of relative leptin resistance at the level of nervous central system. As explained before, although the effects of leptin can favour survival in adverse conditions such as fast, it induces immune alterations blocking the precursor of Treg in favour of the Th17 clone. Adipocyte-derived IL-17 plays a crucial role in the development of chronic inflammation, autoimmunity, insulin resistance [60] and, in our opinion, in the promotion of AD. Leptin interferes with insulin signalling and in type 2 diabetes plasma leptin levels were found to be correlated with the degree of insulin resistance; therefore, insulin resistance syndrome is accompanied by hyperleptinemia as well as hyperinsulinemia [61, 62]. In obese patients leptin and TNF-*α* induce endothelial dysfunction and oxidative stress [63]. Only in body mass of lean individuals leptin regulates insulin action in the peripheral circulation, decreases brain beta-secretase levels and modulates A*β* turnover [64]. Severe obesity is depending on a lack of leptin signalling due to mutation of leptin itself (ob/ob) or the leptin receptor (db/db) resulting in an increase of food intake concomitant with a reduction of energy expenditure. The main mechanisms of leptin resistance previously described are (i) leptin failure to cross the blood-brain barrier because a downregulation of leptin transporter (as LepRa or LepRe), (ii) hypothalamic LepRb downregulation, (iii) abnormalities in the leptin receptor signalling pathways, as inhibition of the JAK2-STAT3 pathway, overexpression of SOCS-3, impairment of PI3K-mTOR pathway or more recently of the ERK pathway [59].

So therefore, hyperleptinemia is a sign of leptin-resistance and this leptin resistant state was associated with impaired activation of the PI3K/AKT pathway and a hyperstimulation of mTOR pathway [65].

As mentioned before, VF secretes more cytokines than subcutaneous adipose tissue and as obesity takes place, several proinflammatory factors in adipose tissue are produced. Moreover, adipocytes size is an important determinant of leptin synthesis, since larger adipocytes contain more leptin than smaller ones [66]. Therefore, summarizing we can say that local inflammation triggered by macrophage accumulation results in numerous metabolic dysfunctions that accompany obesity and bring to the development of systemic inflammation [67]. As evidenced by the work of De Rosa et al. [50] high levels of leptin put Treg cells in an anergic state, leading to the activation of Th1 cells and the release of several inflammatory mediators with a development of that chronic inflammatory state repeatedly reported in obese patients.

As outlined before, though the portal circulation the cytokines reach the liver, where they can stimulate hepatic inflammation thereby inducing a chronic systemic inflammatory response and release of toxic FFA. FFA have long been known to produce deleterious effects on pancreatic beta-cell function inhibiting insulin production and inducing insulin resistance [68] whereas in parallel proinflammatory cytokines, such as TNF-*α*, alter insulin receptors [69].

### 2.7. Adipokines and AD: Protective Role of Leptin

Recent reports have shown that in addition to its action on the hypothalamus, leptin may also exert its effect on the cortex and on the limbic areas, which are involved in cognitive and emotional regulation of feeding behavior [70].

Leptin roles on brain structure and function are being extensively characterised by studies showing that human brain is highly neuroplastic and depends on leptin for its proper development. Additional studies in different populations need to confirm the role of leptin as a biomarker for neurodegenerative diseases.

Some evidence links adipokines directly to cognition. The adipoinsular axis—with leptin and insulin as its main components—has central roles on the regulation of brain function [71]. Leptin regulates food intake and energy metabolism binding to specific regions of the hypothalamus. Recently it has been shown that leptin has extra-hypothalamic effects that may protect the brain against the development of mood and neurodegenerative disorders, such as AD [70, 72]. Leptin appears to exert important effects on brain development as leptin-deficient rodents display abnormal brain development and leptin actively participates in the development of the hypothalamus [73] and in the processes of learning and memory, especially during aging: it was actually described a specific effect in the CA1 region of the hippocampus, selectively altered in AD [74]. Leptin is a potent neurogenic factor not only to hippocampal but also to cortical neurons [75] and has neuroprotective actions against glutamatergic cytotoxicity and oxidative stress [76]. In addition, leptin was shown to promote the proliferation of neuronal precursors as observed following intracerebroventricular administration of a lentiviral vector encoding leptin. After 3 months of treatment the number of proliferating hippocampal cells was increased, as judged by morphometric analysis and by the attenuation of A*β*-induced neurodegeneration [77]. By decreasing the accumulation of intraneuronal lipids, leptin suppresses amyloidogenic pathways. In addition, by inhibiting GSK-3b (the most relevant tau kinase), leptin reduces protein tau phosphorylation, inhibiting the formation of neurofibrillary tangles. The inhibitory effects of leptin on the formation of senile plaques and neurofibrillary tangles seem to be mediated by the selective activation of AMPK in neurons. Leptin was previously shown to reduce the amount of extracellular A*β*, both in cell culture and animal models and its chronic administration resulted in a significant improvement in the cognitive performance of transgenic animal models [78]. In AD, weight loss often precedes the onset of dementia and the level of circulating leptin is inversely proportional to the severity of cognitive decline. It is speculated that a deficiency in leptin levels or function may contribute to systemic and CNS abnormalities leading to disease progression. Furthermore, leptin deficiency may aggravate insulin-controlled pathways, known to be aberrant in AD [78]. As a matter of fact, significantly lower plasma levels of leptin in AD patients compared to the controls were detected [79].

More recently, low leptin levels have been implicated as a direct cause of cognitive impairment, particularly AD [79]. In that case, the absence of beneficial effects of leptin in the central nervous system would predispose to cognitive impairment. However, the protective effect of leptin against the development of AD was observed only among lean individuals; on the contrary obese humans, despite having high leptin levels, may not benefit from protective effects of leptin because of central leptin resistance. In this way a paradoxical situation takes place: leptin is a neuroprotective factor, counteracting AD cognitive impairment, as confirmed by the clinical observation that a weight loss precedes AD manifestations and is accompanied by reduced serum levels of leptin. On the other hand, obese patients exhibit high levels of leptin that cannot perform their protective effects since leptin resistance has been induced at the level of CNS. Other studies are needed to elucidate the molecular mechanisms promoting leptin resistance.

### 2.8. Insulin Resistance in AD

Brain glucose metabolism was found to be impaired in AD [80] and the Rotterdam study and others that followed [81, 82] established that type 2 diabetes mellitus increases the risk for developing cognitive impairment and dementia in AD. In that case, insulin resistance and low insulin levels in the CNS (interestingly referred as *‘‘diabetes of the brain'*') would lead to the accumulation of A*β* and cognitive impairment. Cerebrovascular and central inflammation would contribute further to the pathogenesis of AD [72, 83]. As reported by Hölscher in 2011 [84], a common observation for type 2 diabetes and AD is the desensitization of insulin receptors in the brain. Insulin acts as a growth factor in the brain and shows neuroprotective properties, activating dendritic sprouting, regeneration, and stem cell proliferation. The impairment of growth factor signalling such as early insulin receptor desensitization has been suggested to be involved in the cascade of neurodegenerative events leading to AD [80, 84]. Recently animal models that reflect the pathologic conditions of both type 2 diabetes and AD, were generated. APP23 transgenic mice, a well-established animal model for AD were crossed with ob/ob mice or polygenic NSY mice, as a model for diabetes. Taking advantage of this experimental model, it has been demonstrated that a diabetic condition enhances cognitive dysfunction with cerebrovascular changes such as vascular inflammation and cerebral amyloid angiopathy and that neuropathological changes are associated with impairment of brain insulin signaling [83].

In addition, low insulin levels and insulin resistance can contribute to a decrease in acetylcholine levels, which represents a possible biochemical link between diabetes mellitus and AD [85, 86].

Human and experimental animal studies revealed that neurodegeneration associated with peripheral insulin resistance is likely mediated via a liver-brain axis whereby toxic lipids, including ceramides, cross the blood brain barrier and cause brain insulin resistance, oxidative stress, neuroinflammation, and cell death [87]. Recent evidence demonstrates that sphingolipid metabolism is dysregulated in obesity and specific sphingolipids may provide a common pathway that link excess nutrients and inflammation to increased metabolic and cardiovascular risk [88]. Insulin resistance promotes lipolysis, and lipolysis generates toxic lipids, that is, ceramides, which further impair insulin signalling, mitochondrial function, and cell viability [89]. Cytotoxic ceramides cause insulin resistance by activating pro-inflammatory cytokines and inhibiting insulin-stimulated signalling through PI3 kinase-Akt [90].

### 2.9. Stress and Leptin

As described above, we may argue that obesity itself is a known risk factor for AD, especially in the presence of psychological stress. It is well known that people with depression, especially older adults, have reduced cognitive performance. In addition, many people with dementia also have depression. This illness is associated with elevated levels of cortisol and cytokines which may directly damage the hippocampus and increase the risk of dementia and depression [6, 91]. Depression is frequently a prodrome of dementia and the incidence of depression among patients with AD is estimated to be greater than 40% [92].

Social stressors have effects on food intake and adiposity and, in this case, the individuals with psychological stress have elevated plasma insulin and leptin concentrations compared to nonstressed humans [93]. Glucocorticoids (GC) and insulin interact in the upregulation of serum leptin concentrations. In presence of psychological chronic stress GC lead to overeating and to obesity in spite of elevated leptin concentrations [94]. When the stressor is viewed as a threat without resources to change the coping well with, the stress response is the HPA axis activation and it is a potent trigger of cortisol release [95]. Social stressors have various effects on food intake and adiposity: for example subordinate rats show elevated plasma insulin and leptin concentration compared to dominant animals [96]. Increased GC concentrations have been associated with VF accumulation and with insulin resistance as well as leptin resistance [97]. In humans, the co-elevation of insulin and cortisol is depending from comfort food preference (high fat and sweet food). Palatable comfort food promotes dependence activating brain reward system comprising opioids, dopamine and endocannabinoid. Leptin resistance produces impaired “brake” that in part explain the epidemic “globesity” of eating without metabolic need [98]. The relationship between stress and food intake in humans may also involve effects of GC on NPY, CRH, leptin as well as opioids. It is worthwhile to note that GC receptor density is increased in VF compared to SF and stimulates lipolysis in the whole body [99, 100].

Cortisol increase in presence of insulin inhibits lipid mobilization and promotes the differentiation and proliferation of adipocyte. Increased GC concentration has been associated with insulin and leptin resistance. Those adiposity signals play a role not only in energy regulation but also on the brain reward system by continued search for additional comfort food [101]. Stress-induced cortisol exposure may impair right prefrontal cortex activity, thus impeding the more reflective cognitive control over eating [102]. Therefore, leptin stimulation caused by GC promotes “leptin resistant” obesity and, in turn, obesity may contribute to the reduction of cognitive skills observed in AD. Results from other published studies demonstrate an association of obesity with deficits in learning, memory, and executive functioning in human patients [103, 104]. This relationship between obesity and cognitive impairment has also been documented in experimental animals [105–107]. Collectively, results from the study of Pistell and colleagues reinforce the link between diet-induced obesity and cognitive loss and suggest potentially causal roles for high levels of dietary fats and increased brain inflammation in driving obesity-induced cognitive disruption [108].

Prolonged exposure to proinflammatory cytokines impairs synaptic plasticity, contributing to cognitive and mood disorders [109]. TNF-*α* and IL-1*β*, whose receptors are specifically present at the level of hypothalamus, hippocampus and cortex, were shown to impair neuronal plasticity.

Notably, recent collective reports indicate that after brain injury and in neurodegenerative disorders neurogenesis is controlled by cytokines, chemokines, neurotransmitters, and reactive oxygen/nitrogen species (ROS), which are released by dying neurons as well as by activated macrophages, microglia, and astrocytes [110].

### 2.10. Age-Related Conditions That Can Be Largely Prevented or Delayed by Lifestyle Interventions

#### 2.10.1. Nutritional and Dietary Factors

The studies reported above strongly suggest that alterations of energy metabolism in favor of VF accumulation promote insulin resistance and a chronic inflammatory status which have been recognized as important cofactors in AD initiation and progression. From this, it follows that a preventive strategy should include a reduction of abdominal fat deposits, through a proper nutrition tailored to the individual needs. [12]. Great importance in this respect are showing the modern technologies for analysis of body composition that determine fat mass, fat free mass, total body water, intra- and extracellular water, mineral, metabolism and inflammatory status in the body (BIA-ACC and TomEEx devices) [111, 112]. In fact, thanks to these noninvasive and low cost technologies, it is possible to acquire information on the above parameters and follow up the changes induced in the low level inflammatory status secondary to modifications in lifestyle, especially in diet and physical activity [113–115].

Several follow-up studies have already reported that decreased AD risk is associated with increasing dietary or supplementary intake of antioxidants (e.g., vitamins E and C, fruits and vegetables). A diet high in antioxidants may reduce inflammation, which is associated with the risk of dementia [92]. A variety of dietary supplements have been reported to be beneficial for learning in animals and humans [116]. Positive effects on brain function have been reported for fish oil, teas, fruits, folate, spices, and vitamins [117]. Particularly interesting are plant-derived products such as grapes, blueberries, strawberries, tea, and cocoa, which benefit memory in rodents [118]. Furthermore, studies found that higher adherence to “Mediterranean diet” (i.e., a dietary pattern with higher intake of fish, fruits, and vegetables rich in antioxidants) produced beneficial effects in AD patients. On the contrary diets enriched with saturated fats and cholesterol increase the risk, which is reversed by polyunsaturated fatty acids and fish. Fatty acids may also play a part in the synthesis and fluidity of nerve cell membranes, in synaptic plasticity and neuronal degeneration. In addition, oxidative stress is one of the central features in the AD brain. Thus, it may be plausible that supplementation or diet rich in antioxidants such as fruits, vegetables, and vitamins E and C might protect against AD.

B vitamins were particularly investigated in clinical studies with the attempt to define an association between serum levels of these vitamins and the risk of dementia and AD and different intervention trials were made obtaining, unfortunately, discordant results. As a matter of fact, the Cochrane systematic review concluded that folic acid and vitamin B12 supplementations have no benefits on cognition, although folate plus vitamin B12 are effective in reducing plasma homocysteine.

Folate (vitamin B9), vitamin B12, and vitamin B6 are cofactors in the reactions responsible for homocysteine (Hcy) transformation and removal in the “so called” one-carbon metabolism [119]. Plasma levels of these three vitamins are therefore strictly related to Hcy levels; according to a general paradigm, high plasma Hcy (hyperhomocysteinemia, HHcy) is related to low B vitamin levels. After many studies in humans and in animal models, it is now accepted that moderate HHcy is a potential risk factor for AD, although the possibility that it represents just a marker of the process exists, the association of low B vitamins with AD is still more controversial [120]. However, the majority of retrospective studies evidenced increased Hcy levels in AD subjects and prospective studies pointed out that HHcy is evident before AD development, stressing the idea of a causative function of HHcy in AD [121].

On the basis of these considerations, few interventional studies were performed to evaluate the potentially beneficial supplementation with B vitamins in the attempt to lower Hcy levels and improve cognitive functions. Only one of these studies demonstrated that Hcy-lowering intervention was associated to the improvement of the effects of cholinesterase inhibitors therapy; other studies demonstrated that B vitamin supplementation was able to reduce Hcy levels but did not improve cognitive status or delay cognitive decline [122]. The apparent controversial results obtained after observational and interventional studies in AD patients are probably due to undisclosed biases and methodological troubles occurring in the design of these protocols. Firstly, the most evident (as well as incomprehensible) aspect of the interventional studies on B vitamin supplementation conducted so far is that subjects recruited for the trials had normal Hcy levels; in some case, HHcy was among exclusion criteria. We should suppose that individuals with normal Hcy levels do not present any alteration in one-carbon metabolism; therefore, B vitamin supplementation can very unlikely result in evident beneficial effects. The second aspect to be taken into account is the duration of the trial, since quite short treatments were done in some of these studies. Moreover, the high variability in the doses used for the B vitamin supplementation makes difficult to compare the results obtained from different trials. Finally, appropriate (sensitive) cognitive tests able to reveal subtle differences and larger (more variable) populations could improve the results of the interventional trials with B vitamins supplementation in AD.

#### 2.10.2. Physical Activity

The risk for dementia and AD was also increased in older people with increasing social isolation and less frequent and unsatisfactory contacts with relatives and friends. In fact several studies indicate that a poor social network or social disengagement is associated with cognitive decline and dementia. Rich and large social networks also provide affective and intellectual stimulation that could influence cognitive function and different health outcomes through behavioral, psychological, and physiological pathways [123].

It has also been shown that low-intensity activity such as walking may reduce the risk of dementia and cognitive decline and experimental studies in animal models have established a direct correlation between physical exercise and neurogenesis, especially in the hippocampus [124]. Physical activity is important also in promoting brain plasticity, and it may also affect several gene transcripts and neurotrophic factors that are relevant for the maintenance of cognitive functions. A strong protective effect of regular physical activity in middle age against the development of dementia and AD in late life was reported, especially for persons with the APOE4 allele.

So regular exercise and intellectual stimulation may represent those useful mild stresses that should stimulate maintenance and repair pathways and cause adaptation of cells and the ability to tolerate stronger stresses [125].

An important role in influencing the life span has been attributed to the tissue pH which is related to the metabolites of various nutrients. In a recent study dealing with the yeast chronological life span model it has been shown that acidification of the medium accelerates yeast aging [126]. A central player of this effect has been identified by TOR pathway: when the cell cycle is blocked but mTOR is still active, it causes hypertrophic, hyperactive, hyperfunctional phenotype, with compensatory resistance to signals such as insulin and growth factors, switching quiescence into senescence; therefore, TOR limits life span by accelerating age-related diseases [125]. By contrast, the deletion of either TOR1 or SCH9/S6K seems to extend yeast chronological life span in part by depleting ethanol and acetic acid [127]. These updates support the important role of tissue pH, which should not be acidified by foods high in protein (meat, cheese). “Anti-inflammatory” foods, such as diets rich in fruits and vegetables, may prevent osteopenia and cachectic obesity with an important buffer action and possibly at negative PRAL (potential renal acid load).

## 3. Conclusion

The schematic diagram (Figure 2) illustrates our suggested link between stress, obesity, and Alzheimer's Disease. At the center of the system a series of events, that begin and are maintained by psychosocial stress, trigger the inflammation due to immunological dysregulation that arises from dysmetabolic processes caused by high energy intake.

## Figures and Tables

**Figure 2:**